# The Story of the Insane from Year to Year

**Published:** 1902-10-18

**Authors:** 


### THE STORY OF THE INSANE FROM YEAR TO YEAR.

FIFTEENTH ANNUAL SERIES.

### WEST SUSSEX ASYLUM, CHICHESTER.

FIVE hundred and sixteen patients were in residence at the beginning of the year and 622 at the end of it. There were 168 first admissions, 36 readmissions, 34 discharged recovered, 12 discharged relieved, and 48 deaths. The average number resident was 544, and the total number under treatment was 720. Excluding transfers, the recoveries stand at 25·78 per cent. of the admissions, and the deaths at 8·82 per cent. of the average number resident. Both of these percentages are somewhat low. Of the year's deaths 25 were caused by cerebral and spinal diseases, 2 by consumption, and 5 by other lung diseases. Domestic trouble, mental anxiety, and other moral causes account for the insanity in 21 of the admissions; hereditary influence accounts for 68, previous attacks for 72, and intemperance in drink for 18. The cases sent to Dr. Kidd for treatment were certainly of a very unpromising type, and of the total number of admissions, recovery was considered probable in only 38, possible in 30, improbable in 74, and impossible in 62. The death-rate from phthisis is very low in this asylum, being under 4 per cent. of the total number of deaths. Postmortem examinations were made in 46 of the 48 deaths; but Dr. Kidd merely mentions the bald fact, and does not state what advantages have accrued to himself, or to science, or to the asylum by having so many. A curious incident is related of a female patient, a native of Italy, who was sent under a Home Secretary's warrant to her own country and handed over to the officials of an asylum in Rome. Two months afterwards she found her way back to the West Sussex Asylum. She had escaped from custody, drawn her savings from the bank, and taken a Cook's ticket to England. What makes the incident more remarkable is that her mental condition was so serious that she had to be re-certified and re-admitted next day. New blocks for 300 patients have been opened, and these additions should be sufficient for some years to come; but, alas, there is no finality to asylum extension. It goes on for ever. The committee record " their appreciation of the services rendered by Dr. Kidd in superintending the asylum." The weekly charge to the unions is 12s. 3d. per head.

### WEST RIDING ASYLUM AT WADSLEY, SHEFFIELD.

HERE an increase in the number of patients from 1,652 to 1,670 has to be chronicled. There are 457 first admissions, 83 readmissions, 213 discharged recovered, 76 discharged relieved, 40 discharged not improved, and 193 deaths. The average number resident during the year was 1,660, and the total number under treatment was 2,181. Calculated on the admissions, the recoveries stand at 39·4 per cent.; and calculated on the average number resident, the deaths work out at 11·6 per cent. Of the year's deaths 84 were caused by cerebral and spinal diseases, general paralysis alone accounting for 26 of these; consumption was the cause in 23, pneumonia in 14, bronchitis in 5, pleurisy in 1, and enteritis in 10. Of the year's admissions 238 owed their insanity to moral causes, domestic trouble being the most potent of these; and among the physical causes, intemperance in drink accounts for 81, hereditary influence for 123, congenital defect for 29, and previous attacks for 113. Like so many other medical superintendents, Dr. Kay remarks on the unfavourable nature of the admissions as regards chances of recovery, and he strongly emphasises the advantages and importance of early treatment in insanity. There were two cases of typhoid fever the origin of which could not be traced, and, as already stated, enteritis caused death in 10 instances. As to the latter, Dr. Kay does not say to what he attributes the outbreak. The bacterial treatment of sewage is being tried at this asylum, and we are informed that it has not been so satisfactory as anticipated, and that the effluent is not pure. The visitors say that Dr. Kay and the other medical officers have given every care and attention to the patients. The weekly rate of maintenance is 9s. 4d. per head.

